# Durable efficacy of anlotinib in a patient with advanced thymic squamous cell carcinoma after multiline chemotherapy and apatinib: A case report and literature review

**DOI:** 10.1111/1759-7714.13658

**Published:** 2020-09-30

**Authors:** Ran Zuo, Cuicui Zhang, Li Lin, Zhaoting Meng, Yajie Wang, Yudong Su, Mihray Abudurazik, Ye Du, Peng Chen

**Affiliations:** ^1^ Tianjin Medical University Cancer Institute and Hospital Tianjin China

**Keywords:** Anlotinib, antiangiogenesis, apatinib, thymic carcinoma, tyrosine kinase inhibitor

## Abstract

Thymic carcinoma is a rare and highly aggressive mediastinal tumor. Most patients are diagnosed at surgically unresectable stages. Current prospective and retrospective studies have indicated that platinum and anthracycline‐based chemotherapy are the first choice drugs of first‐line therapy for advanced thymic carcinoma. However, there is no optimal treatment after progression for patients who have undergone first‐line and subsequent chemotherapy. Anlotinib, a novel small molecule tyrosine kinase multitarget inhibitor, was approved by the China Food and Drug Administration as a third‐line treatment for advanced non‐small cell lung cancer (NSCLC) in May 2018. Herein we report a case of an advanced thymic squamous cell carcinoma patient harboring *EGFR* exon 20 insertion who had previously received multiline therapy, including chemotherapy, radiotherapy as well as antiangiogenic therapy. Also as an angiogenesis inhibitor, anotinib had controlled his mediastinal mass after failure of the apatinib treatment. To date, over 23 months of progression‐free survival (PFS) and six years of overall survival (OS) have been achieved. Compared with apatinib, the adverse reactions have been mild and tolerable and the patient's quality of life has improved. To our knowledge, this is the first report where anlotinib has been effective in controlling the progression of thymic carcinoma. In the multiline treatment of advanced thymic carcinoma, anlotinib appears to show great potential when utilized as a salvage treatment.

## Graphical abstract

We present the case of a patient with an advanced TC harboring EGFR exon 20 insertion who successively benefited from two antiangiogenic agents after multiline therapy. After the failure of one tyrosine kinase inhibitor, the other was still active and effectively controlled the growth of the lesion.

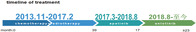

## Introduction

Thymic carcinoma (TC) and thymomas are both thymic epithelial tumors (TET), which are relatively uncommon. The former makes up 15%–20% of thymic tumors,^1^ the incidence rate of which is 1.5 cases per 1 000 000 people every year. Compared with thymomas, TC is prone to local invasion and distant metastasis and has a poor prognosis.^2^ Surgery is the preferred therapeutic modality for early TC. Because of the low incidence and lack of characteristic clinical symptoms, most patients are diagnosed with Masaoka‐Koga stage III or IV. Platinum and anthracycline‐based systemic chemotherapy are the first‐line treatment of choice. However, there is currently no standard treatment for those patients with progressive disease after first‐ or even multiline therapy.

Angiogenesis is essential to tumor genesis and development, which provides oxygen and nutrition, and is regulated by many factors such as vascular endothelial growth factor receptor (VEGFR), platelet‐derived growth factor receptor (PDGFR) and fibroblast growth factor receptor (FGFR), etc.^3^, ^4^ Anlotinib AL3818 is a new oral small molecule multitarget tyrosine kinase inhibitor, which can effectively inhibit VEGFR, PDGFR, FGFR, and c‐kit, etc, with broad‐spectrum antiangiogenic and antitumor efficacy.^5^ Phase III clinical trials suggest that anlotinib exerts a significant effect on patients with advanced non‐small cell lung cancer (NSCLC) with good safety and tolerance. In other clinical trials conducted on different tumors, anlotinib has shown antitumor efficacy. Compared with other TKIs, adverse events (AEs) caused by anlotinib appear to be fewer and less severe, and most AEs are controllable or reversible after medical intervention. Here, we present the case of a patient with an advanced TC harboring EGFR exon 20 insertion who successively benefited from two antiangiogenic drugs after multiline therapy. After the failure of one tyrosine kinase inhibitor, the other was still active and effectively controlled the tumor.

## Case report

This case was reported by our team in 2018, and his previous treatment is detailed in Yudong *et al*.^6^ Here, we provide a detailed report on the patient's follow‐up treatment. A 52‐year‐old Asian male with over 40‐years history of smoking was diagnosed with Masaoka‐Koga stage IVB thymic squamous cell carcinoma at a local hospital. The patient came to us after mutiline chemotherapy (paclitaxel, cisplatin and pemetrexed) and radiotherapy from November 2013 to December 2016, including six cycles of first‐line paclitaxel liposome combined with volumetric intensity‐modulated arc therapy and five cycles of second‐line chemotherapy of paclitaxel and carboplatin as well as six cycles of third‐line pemetrexed and cisplatin. His PFS1 was 23 months, while the PFS2 and PFS3 reached eight and five months, respectively.

**Figure 1:**
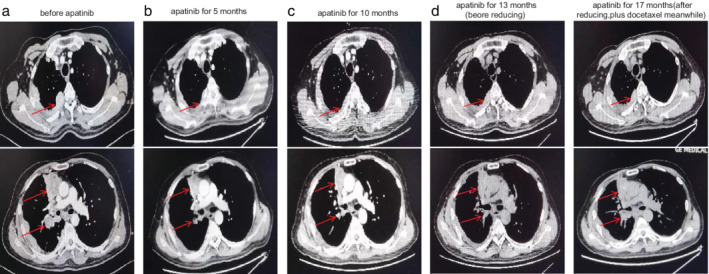
Chest computed tomography (CT) scans before and after apatinib therapy. (**a**) Before apatinib therapy, a soft tissue mass in the mediastinum and obvious multiple metastases in the lymph nodes of the right hilar and mediastinal, right pleura, and right lung were visible. (**b**) After five months of apatinib, chest CT showed a remarkable reduction of the mediastinal mass and metastatic lesions. (**c**) After 10 months of apatinib treatment, the mediastinal mass and metastatic lesions remained stable. (**d**) After 13 months of treatment with apatinib, the mediastinal lesion had progressed, but still remained stable overall. (**e**) After 17 months of apatinib, a chest CT showed progressive disease (PD). The patient had received treatment with apatinib for a total of 17 months.

**Figure 2:**
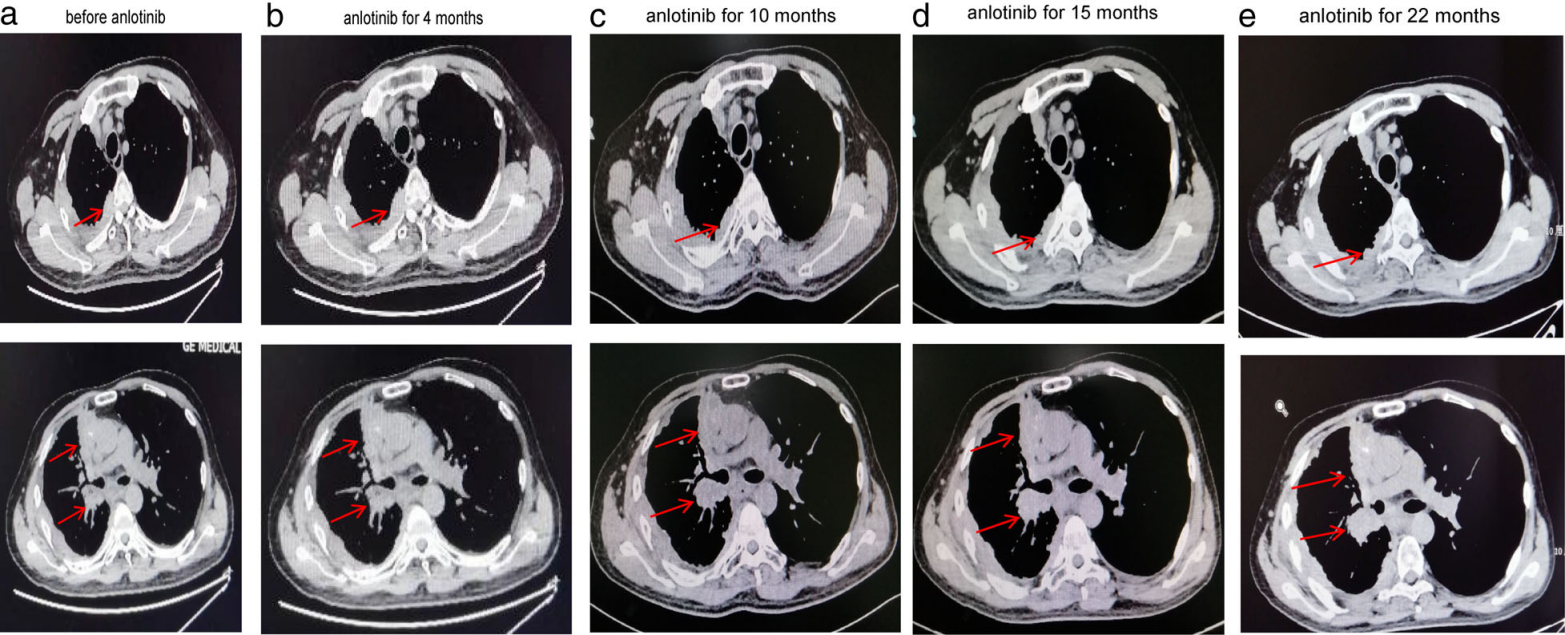
Chest computed tomography (CT) scans before and after anlotinib therapy. (**a**) Chest CT of different layers taken before anlotinib therapy revealed a soft tissue mass in the mediastinum and multiple metastases in the lymph nodes of the right hilum and mediastinal, right pleura, and right lung. (**b**) After four months of anlonitib treatment, chest CT showed that the mediastinal mass and metastatic lesions remain basically stable compared to the baseline. (**c**) After 10 months of anlotinib treatment, there was little change in the mediastinal mass and metastatic lesions on CT compared with the previous scan. (**d**) After 15 months of anlotinib treatment, the mediastinal mass and metastatic lesions remain stable. (**e**) To date, 22 months of PFS have been achieved.

**Figure 3:**
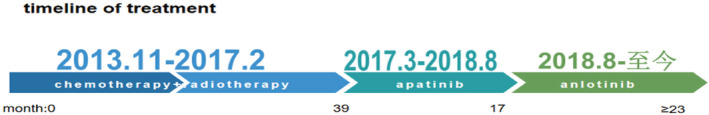
The various treatments the patient received and duration of each treatment.

We completed imaging as well as genomic profiling using next‐generation sequencing (NGS) and supplemented the diagnosis with Masaoka‐Koga stage IVB thymic squamous cell carcinoma accompanied by *EGFR* exon 20 insertion, with a negative PD‐L1 expression. After comprehensive consideration, the patient was administered apatinib, an oral angiogenesis inhibitor, 850 mg/day. The mediastinal mass became smaller in comparison to the baseline after five months and the adverse reactions were tolerated. Regrettably, the mediastinal lesion progressed after 13 months of duration of response and the patient suffered diarrhea and hand‐foot syndrome of grade 3. We tried reducing apatinib to 425 mg/day plus docetaxel (75 mg/μdL), but the regimen was only administered for four cycles when a chest computed tomography (CT) scan in August 2018 confirmed progressive disease (PD). Until August 2018, the patient had taken apatinib for 17 months (Fig 1). Taking into consideration previously administered multiline chemotherapy, antiangiogenic therapy and the patient's relatively long response to apatinib, we decided to administer anlotinib, a novel small molecule tyrosine kinase multitarget inhibitor reported to have less severe adverse reactions. The patient provided their informed consent to treatment with anlotinib. To our amazement, at a dose of 12 mg, once daily on days 1–14 of a 21‐day cycle (two‐weeks on, one‐week off), the mediastinal lesion remained stable. The physical condition of the patient had obviously improved with slightly bleeding gums and diarrhea which required no medical intervention. The patient suffered from grade 2 hypothyroidism with moderate fatigue in May 2020. Under drug intervention (Levothyroxine sodium, 50 μg, take orally, daily), his symptoms of fatigue improved, with FT3 and FT4 increased after one month. The patient has currently been recommended to take Levothyroxine sodium of 75 μg daily according to symptoms and thyroid function without anlotinib reduction. Until now, the patient has achieved a PFS of over 23 months (Fig 2). The treatment has been well tolerated with no obvious adverse reactions. The treatment timeline is shown in Fig 3.

The patient has signed his written informed consent for publishing the case details and the hospital ethics committee approved the report.

## Discussion

Thymic carcinoma is a mediastinal malignant tumor originating from thymic epithelial cells, which is highly aggressive with a poor prognosis. Thymic carcinoma has no specific clinical symptoms in the early stage. Most patients are diagnosed at an advanced stage when the carcinoma is accompanied by multiple metastases, and the best opportunity for surgical resection has been lost.^7^ Despite a 25%–50% response rate to chemotherapy or radiotherapy for patients with advanced TC,^8^ the majority of patients relapse within one year after initial treatment. Because of its rarity and heterogeneity, there is currently no optimal treatment after disease progression for patients who have undergone first and subsequent therapy.

Nowadays, clinicians pay great attention to angiogenesis as it plays a pivotal role in neoplasia and progression. Anlotinib is a new oral small molecule multitarget tyrosine kinase inhibitor, which can strongly inhibit signal pathways mediated by VEGFR, PDGFR and FGFR as well as block tumor angiogenesis completely. Among these receptors, VEGFR2 plays a major role in regulating angiogenesis.^9^ Recently, studies have reported that VEGFR3 play a main role in lymphangiogenesis.^10^ Therefore, the inhibition of both VEGFR2 and VEGFR3 signal pathways may contribute greatly in restraining tumor grow and metastasis. Studies have previously determined that VEGF‐A, VEGFR1 and VEGFR2 are overexpressed in thymomas and TC.^11^ Recently, a prospective phase 2 REMORA trial of an antiangiogenic drug in patients with advanced TC published its results. For patients with advanced TC who progressed after cisplatin‐based chemotherapy, Lenvatinib, a multitargeted inhibitor that potently targets VEGFR1‐3 and FGFR1‐4, c‐kit and other kinases, has shown moderate clinical effect and acceptable toxicity profile.^12^ Sunitinib can inhibit angiogenesis by simultaneously blocking the VEGFR, PDGFR and KIT signaling pathways. A phase II trial of sunitinib in patients with thymic carcinoma who had previously experienced chemotherapy failure reported that the overall response rate (ORR) and disease control rate (DCR) was 26% and 91%, respectively, with median PFS 7.2 months.^13^ In a retrospective study, where sunitinib was applied as a multiline treatment for thymic carcinoma from the French RYTHMIC network, ORR was 20% and median PFS (mPFS) was 3.3 months.^14^ Xie *et al*.^15^ reported that anlotinib showed high selectivity for VEGF family members, especially VEGFR2 and VEGFR3, with IC50 values of 0.2 and 0.7 nmol/L, respectively. Anlotinib was 20‐fold more potent than sunitinib for inhibition of VEGF‐2/‐3. Lin *et al*.^16^ found, at the same dose, the angiogenesis inhibition of anlotinib was superior to other similar drugs such as sunitinib, sorafenib and nintedanib. These data support the use of angiogenesis inhibitors, especially anlotinib in advanced TC.

Drug resistance will inevitably occur due to genetic instability and high mutation of tumor cells. Antiangiogenic therapy is a promising therapeutic regimen because of the genetic stability of endothelial cells.^17^ Tyrosine kinase inhibitors block angiogenesis and might be an alternative therapeutic schedule for patients with multiline chemotherapy and target drugs resistance. Apatinib is a highly selective tyrosine kinase inhibitor, which mainly selectively competes with the ATP‐binding site of VEGFR‐2. We chose apatinib because of the lack of enough evidence for the effectiveness of drugs targeted at driver gene mutations and immunotherapy. The tumor reduced in size after the first five months of treatment. However, the patient was unable to tolerate the adverse reactions associated with the treatment and by the 13th month there was a trend of PD. Studies show that in addition to targeting the above mentioned signaling pathways, anlotinib can directly inhibit the proliferation of tumor cells by inhibiting the proliferation of related receptors (c‐kit) and downstream signaling pathway, which has a dual antitumor effect.^15^ Anlotinib was also administered in our patient at 12 mg once daily for a cycle of two weeks on with one‐week off, which was well tolerated and improved the patient's quality of life without significant toxicity. We suspect that patients may benefit from antiangiogenic therapy rather than inhibiting EGFR exon 20 insertions, and there is currently no evidence that anlotinib inhibits *EGFR* mutations. Taking all the above considerations into account, we chose anlotinib for a salvage treatment, which helped the patient achieve a PFS of over 23 months without obvious adverse reactions.

Nowadays, the potential predictive biomarkers of anlotinib are still unclear, and identifying the ideal predictive efficacy biomarkers with which to screen the patient population will maximize the benefits to patients. In NSCLC, Liu *et al*.^18^ found CD31‐labeled activated circulating endothelial cells (aCECs) could be used as a sensitive marker for the efficacy of anlotinib. Another study suggested serum levels of KLK5 and L1CAM had preferable predictive value for anlotinib response in NSCLC patients receiving third‐line treatment.^19^ In addition, Lu *et al*.^20^ showed that the decrease in serum CCL2 level induced by anlotinib was associated with the benefits of PFS and OS in NSCLC. By performing circulating DNA sequencing, Lu *et al*.^21^ identified that the tumor mutation index (TMI) plus IDH1 exon 4 mutation status was an effective predictor. More biomarkers for thymic carcinoma and other tumors still need to be explored further in the future.

To our knowledge, this is the first clinical report where anlotinib has effectively controlled the progression of advanced thymic squamous cell carcinoma, despite the patient receiving multiline therapy including chemotherapy and radiotherapy, as well as antiangiogenic therapy. To date, over 23 months of PFS and six years of OS have been achieved. AEs appear to be manageable and the patient's quality of life has improved. This patient's treatment will continue to be followed‐up and we hope that he will achieve a longer survival. Anlotinib is expected to be a new treatment option for clinicians to treat advanced TC patients who have undergone mutiline therapy. More multicenter, randomized, controlled clinical trails are needed, and finding the ideal predictive efficacy biomarkers to screen the advantaged population is of great importance in the future.

## Disclosure

Ran Zuo, Cuicui Zhang, Li Lin, Zhaoting Meng, Yajie Wang, Yudong Su, Mireayi, Ye Du, Peng Chen declare that they have no conflicts of interest that might be relevant to the contents of this article.
